# A modulation wave approach to the order hidden in disorder

**DOI:** 10.1107/S2052252514022556

**Published:** 2015-01-01

**Authors:** Ray Withers

**Affiliations:** a Research School of Chemistry, Australian National University, Building 1.38, Sullivan’s Creek Road, Canberra, Australian Central Territory 0200, Australia

**Keywords:** modulation wave approach, ordered ‘disorder’, crystalline structure, long-range order, short-range order

## Abstract

The usefulness of a modulation wave approach to understanding and interpreting the highly structured continuous diffuse intensity distributions characteristic of the reciprocal spaces of the very large family of inherently flexible materials which exhibit ordered ‘disorder’ is pointed out. It is shown that both longer range order and truly short-range order are simultaneously encoded in highly structured diffuse intensity distributions. The long-range ordered crystal chemical rules giving rise to such diffuse distributions are highlighted, along with the existence and usefulness of systematic extinction conditions in these types of structured diffuse distributions.

## Abstract 2

A modulation wave approach is used to interpret the coupled longer range and truly short-range order hidden in the highly structured diffuse intensity distributions of three representative such systems. Both the longer range order and the truly short-range order simultaneously encoded in such highly structured diffuse intensity distributions are highlighted.

## 1. Introduction   

**Figure 1:**
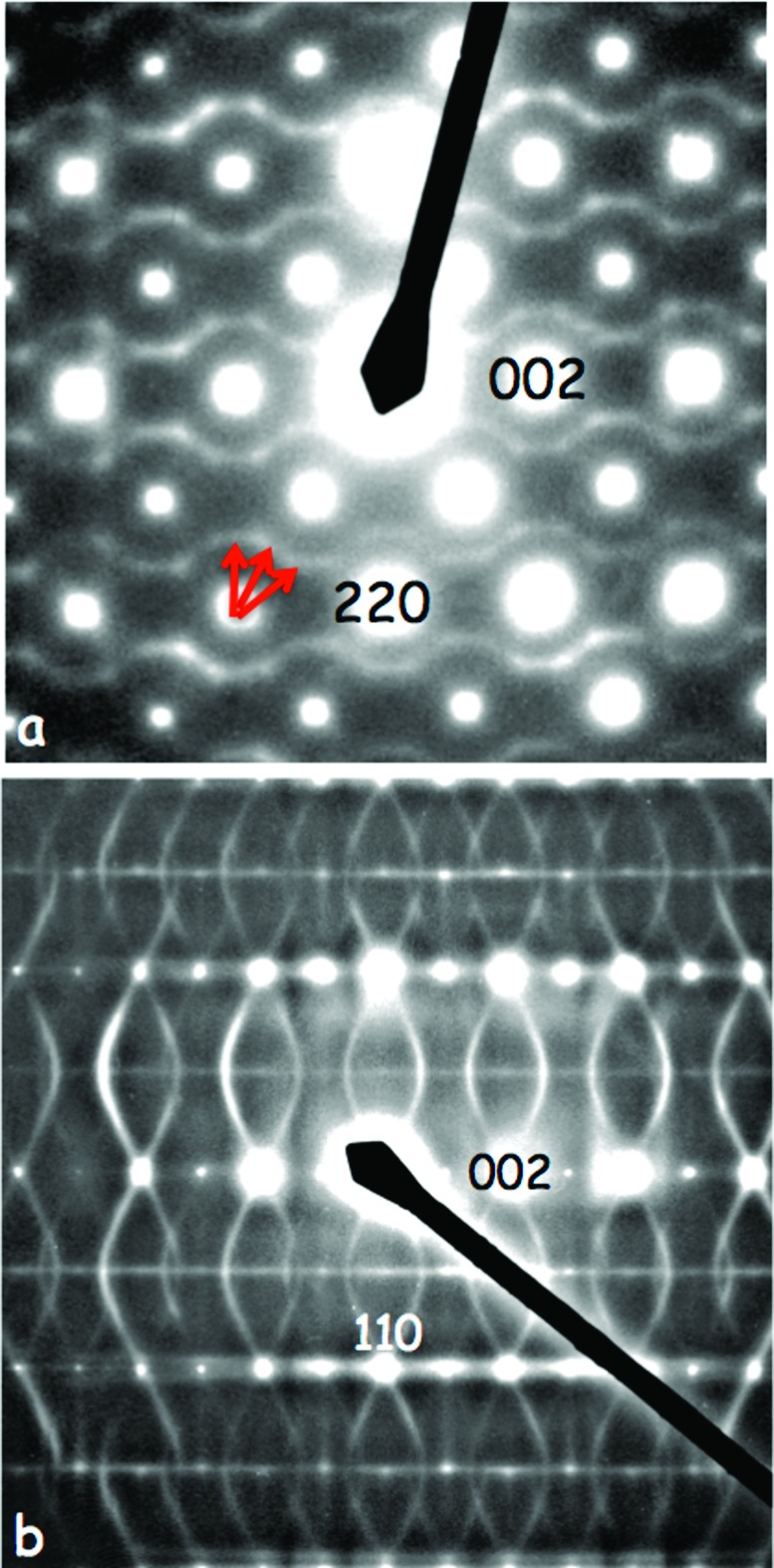
Room-temperature 〈
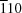
〉 zone-axis EDPs of (*a*) K_3_MoO_3_F_3_ and (*b*) SiO_2_-tridymite above 250°C. The red arrows in (*a*) show some of the independent ‘primary’ modulation wavevectors required to trace out the observed diffuse distribution around each parent Bragg reflection **G**.

**Figure 2:**
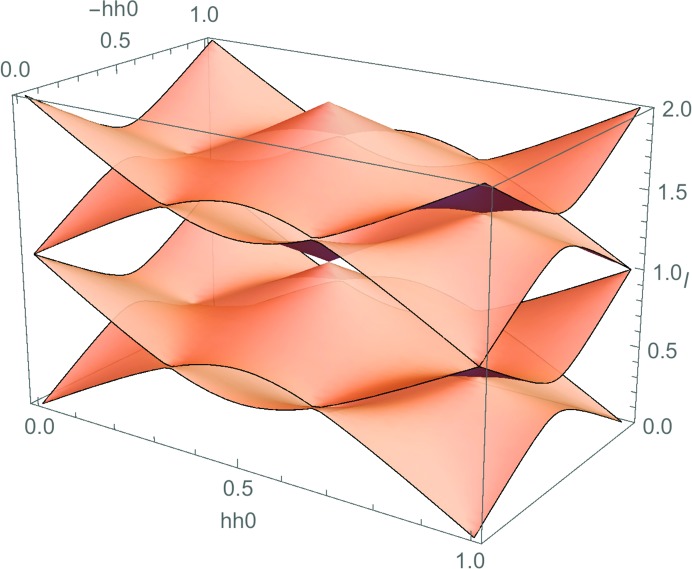
A three-dimensional plot of the sin^2^(π*l*) = 8/9{1 −cos(π*h*)cos(π*k*)cos[π(*h*+*k*)]} (*h, k, l* continuous) curved diffuse intensity characteristic of the *P*6_3_/*mmc* ideal tridymite parent structure. The *hh*0 axis corresponds to the *h* a*+*h* b* axis, the 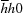 axis to the 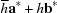 axis and the *l* axis to the *l* c* axis. Rotation of the front face of the plot by 90° around the 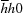 axis enables a direct comparison with Fig. 1 ▶(*b*) above.

In this International Year of Crystallography (IYCr), it seems appropriate to revisit the old question of just what is meant by order and, in particular, disorder, especially among the very widespread class of crystalline materials that exhibit ordered ‘disorder’, *i.e.* materials with reciprocal spaces that are characterized by highly anisotropic structured diffuse scattering accompanying the strong Bragg reflections of an underlying average structure. This is exemplified by *e.g.* the electron diffraction patterns (EDPs) of (*a*) K_3_MoO_3_F_3_ and (*b*) SiO_2_-tridymite above 250°C, shown in Fig. 1 ▶. On the one hand, the existence of continuous diffuse intensity spread out rather evenly across well defined reciprocal space surfaces, *e.g.* sin^2^π*l* = 8/9[1 − cosπ*h* cosπ*k* cosπ(*h* + *k*)] in the case of the highest temperature polymorphic forms of SiO_2_-tridymite shown in Fig. 2 ▶, suggests only truly local short-range order. On the other hand, the existence of local directions in reciprocal space (*e.g.* normal to the continuous diffuse intensity surface shown in Figs. 1 ▶ *b* and 2 ▶) across which the diffuse scattering can really be quite sharp suggests order tending to a much longer range (of the order of tens of nanometres, or more in some cases). What is the meaning of the *shape* of such highly anisotropic structured diffuse distributions (see *e.g.* Fig. 2 ▶)? And how can crystallography provide an appropriate framework to classify and understand the intermediate-range order inherent in such distributions? Clearly, the traditional approach of a (usually limited) set of short-range chemical and displacement atomic pair correlation parameters is not particularly suited to describing well defined reciprocal space diffuse intensity surfaces or the associated implied longer range order.

The traditional notion of order in a crystal is that of a perfectly ordered three-dimensional translationally periodic object, characterized in real space by three-dimensional unit cells and the atoms contained therein, and in reciprocal space by (essentially) infinitely sharp Bragg reflections falling on the nodes of a corresponding three-dimensional reciprocal lattice and their corresponding intensities. It is encapsulated by textbook definitions such as *e.g.* ‘…A unit cell is a (three-dimensional) parallel-sided region … from which the entire crystal can be built up by purely (three-dimensional) translational displacements; unit cells so generated fit together perfectly with no space excluded. …’ (Atkins *et al.*, 2010 ▶). While this three-dimensional definition has worked remarkably well for 100 years or so, there are by now very significant cracks in its generality.

**Figure 3:**
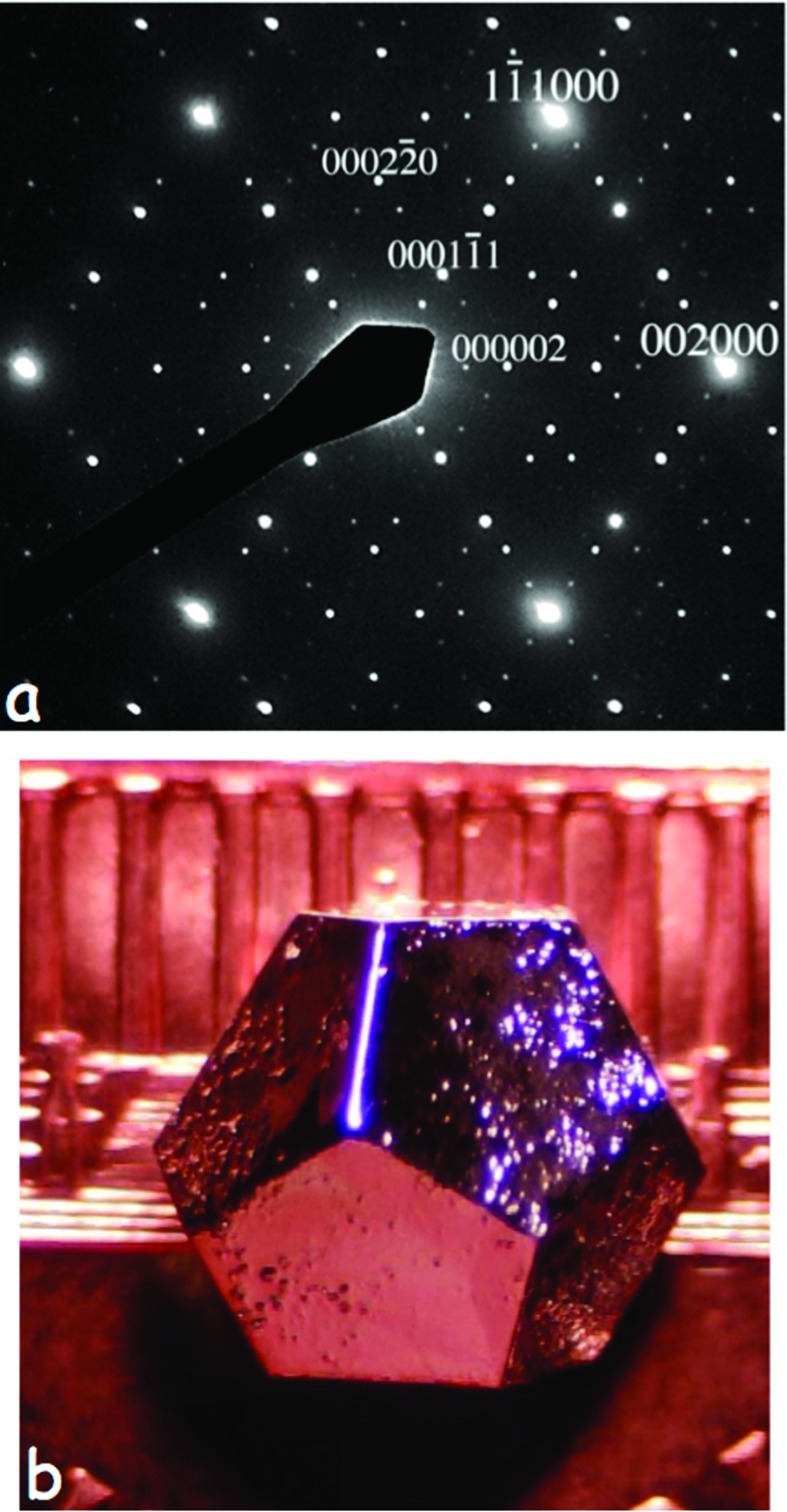
(*a*) A 〈110〉 zone-axis EDP typical of the (3+3)-dimensional incommensurately modulated (1−*x*)Bi_2_O_3_·*x*Nb_2_O_5_ (0.06 ≤ *x* ≤ 0.25) solid-solution phase for *x* = 0.2 [see Ling *et al.* (1998 ▶, 2013 ▶) for details]. (*b*) An optical micrograph of the perfect dodecahedral symmetry of the millimetre-sized single icosahedral quasicrystal Ho_8.7_Mg_34.6_Zn_56.8_ [see Fisher *et al.* (1998 ▶); micrograph courtesy of I. R. Fisher].

The discovery of long-range ordered incommensurately modulated structures in the early 1960s (Herpin *et al.*, 1960 ▶; Brouns *et al.*, 1964 ▶) and composite modulated structures in the 1970s (Johnson & Watson, 1976 ▶; Pouget *et al.*, 1978 ▶), followed by icosohedral, octagonal, decagonal and dodecagonal quasicrystalline phases (Shechtman *et al.*, 1984 ▶; Ishimasa *et al.*, 1985 ▶; Steurer, 2012 ▶) from the early 1980s onwards [see Janssen (2013 ▶) for a good review of the early history of aperiodic crystals], has by now blown quite large holes in the above definition of crystalline order. For example, three-dimensional integer indexing of reciprocal lattices is no longer possible for aperiodic crystals [see *e.g.* Fig. 3 ▶ *a*; see also Ling *et al.* (1998 ▶, 2013 ▶)]. Likewise, the constraint imposed by the original definition that true crystals should never display five-, eight-, ten- and 12-fold rotational symmetries is also clearly no longer necessarily valid [see *e.g.* Fig. 3 ▶ *b*; see also Fisher *et al.* (1998 ▶)], although the reluctance to alter the earlier three-dimensional definition lingers on in the ‘quasi’-crystalline or aperiodic, rather than simply crystalline, name often used for these ordered crystals. In a similar manner, the description of all non-Bragg scattering as diffuse scattering in the original meaning of the word ‘diffuse’, *i.e.* something that does not have a regular shape or is spread out over a large volume, is far too broad and reinforces the need, in this author’s opinion, for a clear distinction between truly short-range order (≲1 nm or so)/genuine ‘diffuse’ scattering, and the rather longer range or intermediate order encoded in the highly structured diffuse intensity distributions shown in Fig. 1 ▶.

The idea of embedding aperiodic crystal structures into higher (3+*d*)-dimensional superspace [see *e.g.* de Wolff (1977 ▶), Janner & Janssen (1977 ▶), Janssen (1986 ▶, 2013 ▶), Baake & Grimm (2009 ▶), and references contained therein] led to the recovery of periodicity in higher dimensional real and reciprocal space, and subsequently to a new broader definition of crystalline order by the International Union of Crystallography (IUCr)’s Commission on Aperiodic Crystals in 1992: ‘…a ‘crystal’ is any solid having an essentially discrete diffraction diagram…’. While a significantly broader notion than the original, this new definition still tends to constrain ideas and thinking as to the nature of order, especially intermediate-range order, well beyond the simple average structure and how best to characterize it (Mackay, 1969 ▶; Mackay & Finney, 1973 ▶; Janner, 1997 ▶, 2001 ▶; Baake & Grimm, 2009 ▶, 2012 ▶). In particular, the definition of a crystal in terms of pure point diffraction/sharp Bragg reflections *only* appears to rule out the unavoidable simultaneous presence of other types of order (in principle, of relatively long range) that are very often present in crystalline materials and are responsible for the highly anisotropic structured continuous diffuse scattering apparent in *e.g.* Fig. 1 ▶. It also hinders thinking about ways in which crystallography might evolve in order to interpret and best characterize the very real additional order inherent in such nominal ‘disorder’.

The purpose of this paper is to highlight the usefulness of a modulation wave approach to interpreting the highly structured continuous diffuse intensity distributions characteristic of the reciprocal spaces of the large family of materials which exhibit ordered ‘disorder’ (see *e.g.* Fig. 1 ▶), to show how it is often possible (in such cases) to separate out the truly local short-range order from the hidden longer range order in such ‘disorder’, and to stimulate further thinking as to the nature of order and disorder.

## 2. Modulated structures and a modulation wave approach to structured ‘diffuse’ scattering   

The very notion of a modulated structure suggests an overall atomic arrangement (structure) that is inherently hierarchical and with a delicately balanced local free-energy landscape: one with a well defined underlying average, or parent, structure, yet with at least some remaining (probably competing) degrees of freedom! In a chemically disordered system, for example, there might be a hard constraint on the local stoichiometry around a particular atom in each average structure unit cell, but still remaining degrees of freedom as regards the relative orientation of that local stoichiometry from one such atom to the next and/or from one unit cell to the next. Indeed, this is the case in two of the three systems discussed below. If the total constraints acting on the system are insufficient relative to the degrees of freedom, a modulated structure of some sort or other is likely to result, either in the form of a commensurate or incommensurately modulated structure with a limited number of independent primary modulation wavevectors **q** (and hence degrees of structural freedom; see *e.g.* Fig. 3 ▶ *a*), or a highly structured continuous diffuse intensity distribution (corresponding to the rather larger number of independent primary modulation wavevectors required to trace out the observed diffuse distribution around each parent Bragg reflection **G**, and with considerably more degrees of structural freedom in the sense that there are many more such wavevectors required; see *e.g.* Figs. 1 ▶ and 2 ▶). In the language of modulated structures, the set of primary modulation wavevectors {**q**_1_, …, **q**_d_} of any modulated structure are simply the supplementary basis vectors required in addition to the conventional three-dimensional basis vectors (a*, b* and c*) in order to integer-index reciprocal space. For further information on the meaning of independent primary modulation wavevectors and modulation waves in general, see Perez-Mato *et al.* (1986 ▶, 1987 ▶). For practical examples of how such a modulation wave approach can be practically applied to synthesize plausible real-space structures, see *e.g.* Welberry & Butler (1994 ▶) and Welberry *et al.* (1995 ▶).

A few of the suggested independent primary modulation wavevectors are shown by the red arrows in Fig. 1 ▶(*a*). This may seem a drastic approach, given that higher order harmonic satellite reflections and combinations thereof are very commonly observed in truly long-range ordered incommensurate modulated structures (see *e.g.* Fig. 3 ▶ *a*). On the other hand, note that there are no observed higher order harmonic diffuse shapes or combinations thereof observed in either Fig. 1 ▶(*a*) or Fig. 1 ▶(*b*), and that this is the norm for such structured diffuse distributions [see *e.g.* Withers (2008 ▶)]. Indeed, if this were not the case, the well defined shapes of the highly structured diffuse intensity distributions discussed in this paper would not exist. Such observations strongly suggest, indeed probably require, that there is almost invariably never any correlation between the different individual modulation waves with wavevectors constituting the observed diffuse distribution. One way in which this can be achieved is to assume that each individual modulation wave making up the overall structured diffuse distribution contributes to different finite regions of real space, thus giving rise to a diffuse peak in reciprocal space with a finite width corresponding to the width of the observed highly structured diffuse distribution [see *e.g.* Welberry & Butler (1994 ▶) and Welberry *et al.* (1995 ▶), and references contained therein, for examples of how this can be achieved practically]. In this sense, such regions (which can be on the scale of tens of nanometres or longer) are precursors of the different long-range ordered modulated structures possible. This is the basis of the modulation wave approach to structured diffuse scattering advocated in this paper.

Given this approach, it is now possible to calculate structure factors for the observed diffuse intensity at **G** ± **q**, where **q** in this context represents the entire set of independent primary modulation wavevectors required to trace out the observed diffuse distribution around each parent Bragg reflection, **G**, using the same approach as that used for normal modulated structures, except that the intensity at **G** ± **q** is only calculated up to third order in the correponding compositional and displacive modulation wave amplitudes [see *e.g.* Withers (2008 ▶)].

### 2.1. Structure factor expressions and the modulation wave approach   

If we were dealing with a conventional incommensurately modulated structure, rather than a material exhibiting a highly structured continuous diffuse intensity distribution, we would start with an underlying average or parent structure (characterized by a set of typically strong average-structure Bragg reflections **G** = *h* a* + *k* b* + *l* c*) and a limited set of *s* = 1–*d* (*d* typically = 1, 2 or 3 to date) additional primary modulation wavevectors **q**_*s*_ from which all other observed satellite reflections at **G** ± Σ_*s*=1–d_ *m*_*s*_ **q**_*s*_ (*m*_*s*_ are integer) could be integer indexed (see *e.g.* Fig. 3 ▶ *a*). However, in our case, experiment (see *e.g.* Fig. 1 ▶) dictates that we do not need any Σ_*s*=1–d_ *m*_*s*_ **q**_*s*_ cross-terms.

In a modulation wave approach [see *e.g.* Perez-Mato *et al.* (1986 ▶, 1987 ▶), Janssen *et al.* (1995 ▶) and Withers (2008 ▶)], the composition modulation wave associated with the modulation wavevector **q**, *i.e.* the deviation of the scattering factor of the μth atom in the primitive average structure unit cell **t** away from its average value, δ*f*_μ_, can be written in the form
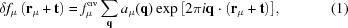
where 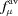 represents the average atomic scattering factor of the μth atom over all unit cells **t**, and *a*_μ_(**q**) represents the complex compositional eigenvector associated with the modulation wavevector **q**, with the property that *a*_μ_(−**q**) = *a*_μ_(**q**)*. The complex compositional eigenvector *a*_μ_(**q**) = *A*_μ_(**q**)exp *i*θ_μ_(**q**), where *A*_μ_(**q**) is the real compositional modulation wave amplitude and θ_μ_(**q**) is the compositional modulation wave phase associated with the modulation wavevector **q**. [For further details on the notation used, see Withers (2008 ▶); see also Perez-Mato *et al.* (1986 ▶, 1987 ▶) and Janssen *et al.* (1995 ▶)]. Likewise, the displacement of the μth atom in the unit cell **t** away from its average structure position at (**r**_μ_ + **t**) can be written
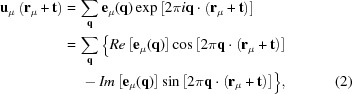
where **e**_μ_(**q**) = Σ_**α = a,b,c**_ **α**∊_μα_exp(*i*θ_μα_) is the complex displacement eigenvector of the μth atom associated with the modulation wavevector **q** and has the property that **e**_μ_(−**q**) = **e**_μ_(**q**)*. Note that *a*_μ_(**q**) is a scalar while **e**_μ_(**q**) is a vector.

The total scattering amplitude, *F*(**k**), from such a modulated structure can then be written as
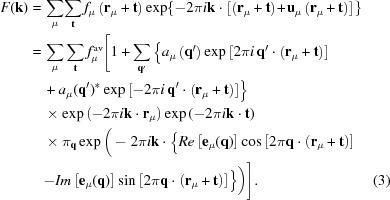
The minus sign in the exp(−2π*i* **k**…) factor in equation (3) is not standard crystallographic usage, but it is used here to be logically consistent with the use of + signs in the exp(2π*i* **k**…) factors in equations (1) and (2) above.

Then, using the Jacobi–Auger generating relation exp(*ix*sinθ) = Σ*_m_J_m_*(*x*)exp(*im*θ) (where the summation over the integer *m* is from −∞ to ∞ and *J_m_* are *m*th-order Bessel functions), coupled with the further assumption that the generally weak primary diffuse distribution at **G** ± **q** arises only from compositional and/or displacive modulation waves associated with the different individual primary modulation wavevectors **q**, supplemented (in more anharmonic cases) by the addition of necessarily small-amplitude compositional and/or displacive modulation waves associated with the second-harmonic modulation wavevectors 2**q**, it can be shown that
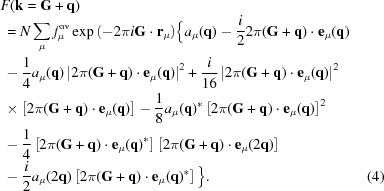
For full details of the derivation, see Withers (2008 ▶). The above expression has been expanded up to third order in the modulation wave eigenvectors *a*_μ_(**q**) and **e**_μ_(**q**). This is the fundamental equation governing the scattering from a dis­ordered modulated structure, and it will be used below to understand and interpret important qualitative and quantitative features of structured diffuse intensity distributions. In practice, for most disordered systems (see *e.g.* Fig. 1 ▶), it is only necessary to include the first two terms (or first line) of the above expression, *i.e.* to include the expansion in equation (4) only up to first order.

## 3. Practical applications of the modulation wave approach to structured diffuse distributions   

With the above structure factor expression, we are now in a position to tackle ordered ‘disorder’ in three systems below, which illustrate the use of the modulation wave approach and the type of information that can thereby be extracted.

### 3.1. The iron oxyfluoride FeOF   

**Figure 4:**
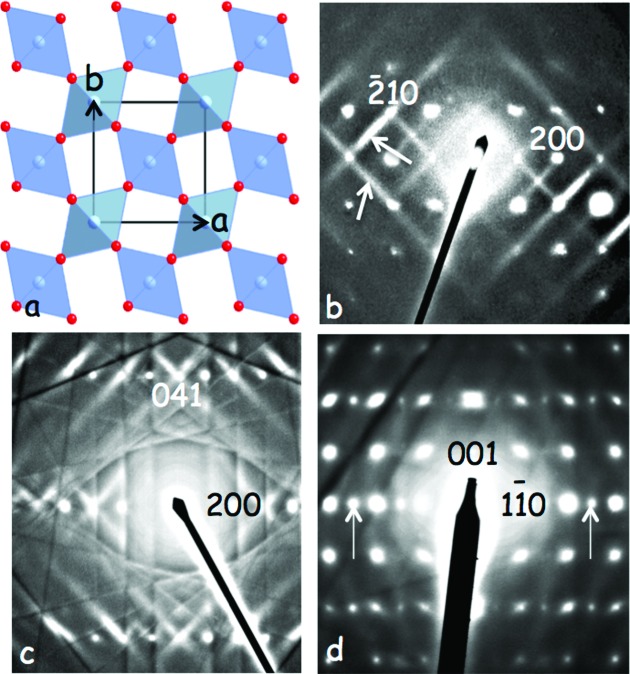
(*a*) The rutile average structure of Fe^3+^OF, in projection along [001] (O/F anions, *A*, are denoted by small red balls and the Fe atoms are in the Fe*A*_6_ octahedra). (*b*) Close to [001], (*c*) 〈
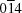
〉 and (*d*) 〈110〉 zone-axis EDPs of FeOF. Note the presence of **G**±∊[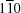]* (∊ continuous) rods of diffuse intensity in (*b*) (indicated by white arrows) but their systematic absence in (*d*). Note also the apparent appearance of **G**±
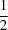
[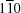]* ‘satellite reflections’ [indicated by white arrows in (*d*)] arising from the **G**±∊[110]* diffuse rods cutting through the ZOLZ.

Consider, for example, the relatively simple case of the iron oxyfluoride, FeOF, with a rutile (average) structure type, space group symmetry *P*4_2_/*mnm* (see Fig. 4 ▶ *a*). Is there any O/F ordering? Conventional crystallography, without taking structured diffuse scattering into account, cannot tell. Mossbauer spectroscopy, on the other hand, is extremely sensitive to the immediate pseudo-octahedral coordination environment of the Fe atoms and finds that there is only one unique Fe environment which must therefore, by necessity, be of O_3_F_3_ type. This is not too surprising and represents an example of an old axiom dating back to Pauling that the smallest building blocks of a compositionally disordered system should each have, as far as possible, the same composition as the overall macroscopic composition in order to minimize substitutional strain. Again, how are the O and F atoms arranged around the local Fe atoms?

For this we have to turn to structured diffuse scattering. Figs. 4 ▶(*b*)–4 ▶(*d*) also show the zone axis EDPs of FeOF close to [001], 〈
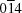
〉 and 〈110〉, respectively. Notice the 〈110〉* transverse polarized rods of diffuse intensity running almost solely through the *h*+*k*+*l* parent reflections in both Fig. 4 ▶(*b*) and Fig. 4 ▶(*c*). In Fig. 4 ▶(*c*), the sample is tilted 21° away from the [001] zone-axis orientation so that the one-dimensional rods of diffuse intensity are no longer in the zero-order Laue zone (ZOLZ) plane but are not that far removed, so that they are visible coming out of the parent Bragg reflections for some distance but eventually fade away. In the case of the 〈110〉 zone-axis EDP shown in Fig. 4 ▶(*d*), the **G**±∊[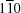]* (∊ continuous) diffuse one-dimensional rods should be in the ZOLZ and hence visible but are in fact systematically absent. The ∊[110]* (∊ continuous) rods are oriented perpendicular to the ZOLZ and hence give the appearance of **G**±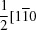]* ‘satellite reflections’ (see the apparent ‘reflections’, arrowed in white, arising from cutting perpendicularly through the one-dimensional rods). It is clear that the reciprocal space of FeOF is characterized by one-dimensional 〈110〉* rods of diffuse intensity running almost solely through the *h*+*k*+*l* parent reflections [for full details, see the original paper by Brink *et al.* (2000 ▶)].

What does this tell us? Firstly, the Fourier transform of a one-dimensional rod in reciprocal space is a two-dimensional plane in real space and *vice versa*, so that the observed 〈110〉* rods of diffuse intensity in FeOF imply longish range ordering (certainly above the range of several nanometres) in the orthogonal 〈110〉 planes of real space, but virtually no correlation from one such 〈110〉 plane of real space to the next in the extended rod direction. (Note that, if the reciprocal space width of the one-dimensional rods could be reliably measured from EDPs such as those shown in Fig. 4 ▶, then the correlation length of the implied two-dimensional order in real space could be determined experimentally. However, the typically weak intensity of structured diffuse distributions often means that incident-beam probes need to be focused somewhat in order to observe the diffuse distribution in a reasonable amount of time. In such a case, as here, only a distinctly lower bound on the actual two-dimensional correlation length can then be reliably measured, as the observed width is convoluted with unknown instrumental parameters, including the convergence angle of the incident probe *etc.* The same considerations apply to all the experimental EDPs in this paper). So FeOF has rather long-range O/F ordering in both the (110) and (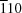) planes, but how? Bond-valence sum calculations [see *e.g.* Brown (2006 ▶)] of the refined average structure give apparent valences (AVs) or bond-valence sums for Fe^3+^ of 2.95 (close to the ideal value of 3), −1.56 for O^2−^ on the anion sites (*i.e.* significantly under-bonded) and −1.22 for F^1−^ on the anion sites (*i.e.* significantly over-bonded). Thus, it makes perfect crystal-chemical common sense that O should always be opposite F in each octahedron in the {110} plane. The Fe atoms can then move off-centre away from the F atoms and towards the O atoms in order to satisfy local crystal-chemistry considerations.

**Figure 5:**
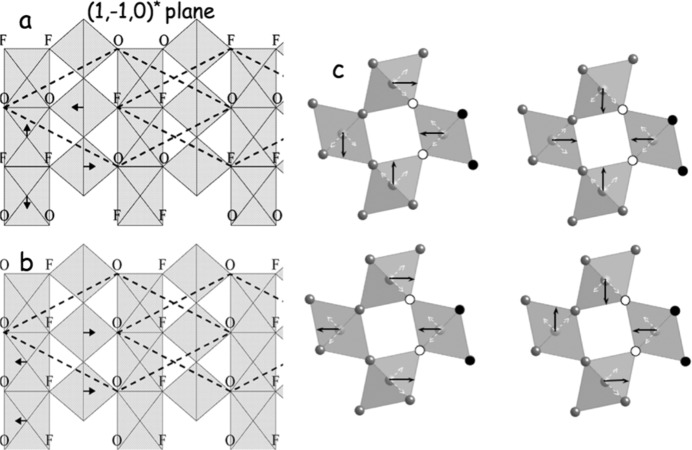
Parts (*a*) and (*b*) show the two distinct planar long-range O/F ordered possibilities, assuming that an O atom is always opposite an F atom in each FeO_3_F_3_ pseudo-octahedron, along with the O/F ordering-induced Fe displacements [of magnitude 0.12–0.13 Å/(2^1/2^) in (*a*) and (*b*), and of magnitude 0.12–0.13 Å in (*c*)]. Note that the [110] direction of rutile is horizontal in (*a*) and (*b*), while the [001] or *c* direction is vertical. Part (*c*) shows the possible O/F ordering patterns in nearest-neigbour clusters of four FeO_3_F_3_ octahedra, assuming that the right-hand octahedron has the same O/F ordering pattern in each case. The F ions for this octahedron are represented by small black balls, while the oxygen ions are represented by small circled white balls. The [110] direction is vertical while the [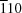] direction is horizontal in (*c*).

There are, however, two distinct planar long-range O/F ordered possibilities, as shown in Figs. 5 ▶(*a*) and 5 ▶(*b*). To distinguish between these two possibilities, we need to use a symmetry argument. Note the presence of the **G**±∊[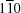]* (∊ continuous) rod of diffuse intensity (arrowed) in Fig. 4 ▶(*b*) but its systematic absence in Fig. 4 ▶(*d*). This characteristic behaviour implies a systematic extinction condition in the diffuse distribution (given by **q** = ±∊[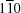]*). Such extinction conditions occur quite commonly in diffuse distributions and should be exploited much more, particularly in cases where the observed diffuse scattering takes a rod-like or planar shape [see *e.g.* Withers *et al.* (2010 ▶)]. In this particular case, the small co-group or symmetry of the modulation wavevector of **q**, for any ∊, is given by **G^q^** = {*E, C*_
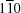
_, *m*_110_, *m*_*z*_} in the notation of Bradley & Cracknell (1972 ▶). The possible O/F ordering pattern in Fig. 5 ▶(*a*) is characterized by an irreducible representation (irrep) with a character of 1 under a mirror plane perpendicular to [110], *m*_110_, and −1 under a mirror plane perpendicular to [001], *m*_*z*_. The consequence is that **G**±∊[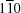]* rods of diffuse intensity should be absent in Fig. 4 ▶(*b*) but present in Fig. 4 ▶(*d*), exactly the opposite of what is observed experimentally. On the other hand, the O/F ordering pattern shown in Fig. 5 ▶(*b*) is characterized by an irrep with a character of −1 under a mirror plane perpendicular to [110], *m*_110_, and 1 under a mirror plane perpendicular to [001], *m*_*z*_. The consequence this time is that the **G**±∊[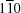]* rods of diffuse intensity will be present in Fig. 4 ▶(*b*) but absent in Fig. 4 ▶(*d*), exactly what is observed experimentally. We therefore know that the O/F ordering in the {110} planes is as shown in Fig. 5 ▶(*b*).

Likewise, there is a Bravais lattice-like pseudo-extinction condition in the diffuse scattering in that, to a very good approximation, 〈110〉* rods of diffuse intensity are only ever observed through *h*+*k*+*l* odd parent reflections. Why should this be the case? Consider the structure factor expression deduced in §2.1 above up to first order in the modulation wave eigenvectors, *a*_μ_(**q**) and **e**_μ_(**q**), as defined in equation (4), *i.e.*

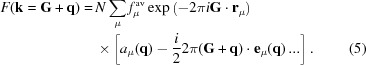
Now, given the strong azimuthal intensity variation displayed by the EDPs shown in Figs. 4 ▶(*b*)–4 ▶(*d*), it is apparent that the Fe displacements dominate the observed diffuse intensity. This is not surprising, given that O neighbours F in the periodic table. The amplitude of the compositional modulation wave is thus vanishingly small. The Fe contribution to the observed diffuse intensity is given by
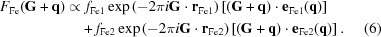
Now, putting the origin on Fe1, **r**_Fe1_ = (0, 0, 0) and **r**_Fe2_ = (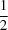, 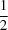, 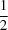), while **e**_Fe2_(**q**) = −**e**_Fe1_(**q**) for all **q** = ∊〈110〉* (see Fig. 5 ▶ *b*). Substitution into equation (6) then gives
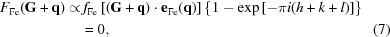
unless (*h* + *k* + *l*) is odd, just as is observed experimentally (see Figs. 4 ▶ *b*–4 ▶ *d*).

Finally, consider Fig. 5 ▶(*c*), which shows the possible O/F ordering distributions in nearest-neigbouring clusters of four FeO_3_F_3_ octahedra. Note that the right-most octahedron in each cluster has the same O/F ordering pattern and induced off-centre Fe displacement in each case. The [110] direction is vertical while the [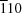] direction is horizontal in Fig. 5 ▶(*c*). There are then four possible such patterns, as shown in Fig. 5 ▶(*c*), which each obey the O/F distribution rule as given in Fig. 5 ▶(*b*) along the two orthogonal {110} planes, and yet not all the degrees of freedom are nailed down, *i.e.* the observed structured diffuse distribution in the case of FeOF shows there is insufficient (or even no!?) enthalpic free energy to be gained by ordering in more than two dimensions. Either the ‘dis­ordered’ state is then truly the ground state or entropy wins!

### 3.2. The ‘defect’ NaCl-type (1−*x*)MgS·*x*Yb_2*x*/3_S = Mg_1−*x*_Yb_2*x*/3_□_*x*/3_S (0 ≤ *x* ≲ 0.45) solid solution   

**Figure 6:**
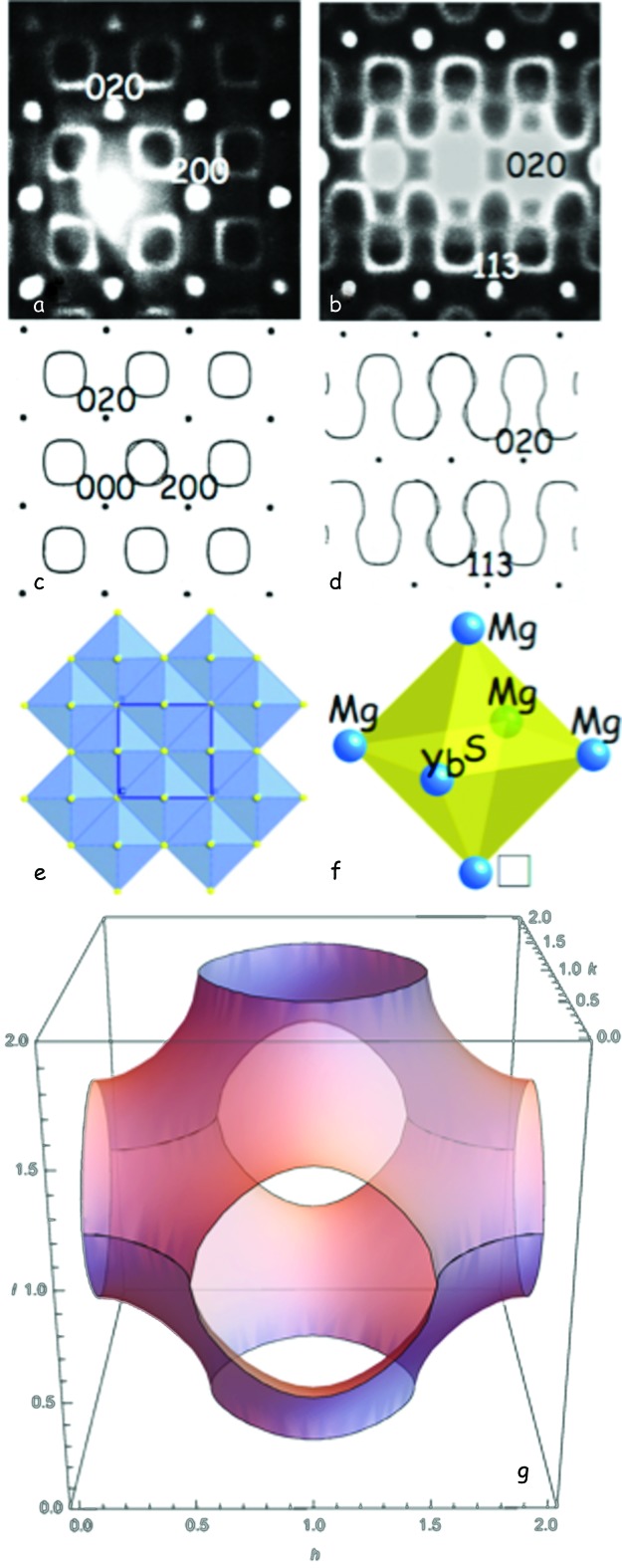
(*a*) [001] and (*b*) 〈
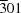
〉 zone-axis EDPs of the disordered defect NaCl-type Mg^2+^_(1−*x*)_Yb_2/3_^3+^□_*x*/3_S (0 ≤ *x* ≤ 0.45) solid-solution phase. Parts (*c*) and (*d*) show equivalent calculated sections through the diffuse surface defined by the equation cos(π*h*) + cos(π*k*) + cos(π*l*) = 0 [shown in part (*g*); here, **q** defining the diffuse intensity surface is written as **q** = *h* a*+*k* b*+*l* c* and *h, k* and *l* are continuous variables] from Sauvage & Parthé (1972 ▶). Part (*e*) shows the defect NaCl-type average structure along an 〈001〉 direction and (*f*) shows the stoichiometrically most common SMg_4_Yb_1_□_1_ local octahedron. The *h* axis corresponds to the *h* a* axis, the *k* axis to the *k* b* axis and the *l* axis to the *l* c* axis in (*g*).

What happens when the diffuse shape is not a simple rod or plane, so that symmetry arguments or irreps, as used above for FeOF, can no longer be used? Consider the case of the ‘defect’ NaCl-type (1−*x*)MgS·*x*Yb_2*x*/3_S = Mg_1−*x*_Yb_2*x*/3_□_*x*/3_S (0 ≤ *x* ≲ 0.45) solid solution [representative of the family of wide-range non-stoichiometric solid solutions; see also Urones-Garrote *et al.* (2005 ▶) and Withers (2008 ▶); here and in what follows, □ represents a vacancy]. Figs. 6 ▶(*a*) and 6 ▶(*b*) show typical [001] and 〈
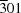
〉 zone axis EDPs, respectively, of the disordered defect NaCl-type Mg^2+^_(1−*x*)_Yb_2/3_^3+^□_*x*/3_S (0 ≤ *x* ≤ 0.45) solid-solution phase, taken for *x* = 0.30. Figs. 6 ▶(*c*) and 6 ▶(*d*) show equivalent calculated sections through the diffuse surface defined by the simple equation cos(π*h*) + cos(π*k*) + cos(π*l*) = 0 (shown in Fig. 6 ▶ *g*), where **q** defining the diffuse intensity surface is written as **q** = *h* a* + *k* b* + *l* c* and *h, k* and *l* are here continuous variables; see also Fig. 3 of Sauvage & Parthé (1972 ▶), and also Billingham *et al.* (1972 ▶).

Where does this equation come from? From Pauling’s very useful axiom discussed above, namely that the smallest building blocks of a compositionally disordered system should each have, as far as possible, the same composition as the overall macroscopic composition in each primitive unit cell, **t**, in order to minimize substitutional strain. In the current case of (1−*x*)MgS·*x*Yb_2*x*/3_S = Mg_1−*x*_Yb_2*x*/3_□_*x*/3_S for *x* = 0.3, this means that each S atom in the smallest octahedral building block, the S*M*_6_ octahedron (see Fig. 6 ▶ *e*), should always be surrounded, as far as possible, by the average number of metal atoms, *i.e* 4.2 Mg atoms, 1.2 Yb atoms and 0.6 vacancies. Clearly, it is not possible to satisfy this exactly locally at any particular average structure unit cell, *i.e.* the value of **t**. However, if the most common, or indeed only, local octahedral configurations are Mg_4_Yb_1_□_1_ (for 60% of the available values of **t**, *i.e.* for 60% of the average structure unit cells; see Fig. 6 ▶ *f*), Mg_5_Yb_1_□_0_ (20% of the available values of **t**) and Mg_4_Yb_2_□_0_ (also for 20% of the available values of **t**) in whatever orientation, then the desired average stoichiometry of Mg_0.7_Yb_0.2_□_0.1_S would result.

Clearly, local short-range order exists in that there are many different local arrangements possible, *i.e.* it is clear that short-range order (SRO) exists and the diffuse distribution should be continuous. But where does the long-range order (LRO), *i.e.* the sharpness of the diffuse distribution, come from? Here the modulation wave approach is invaluable. In the NaCl-type average structure of Mg_1−*x*_Yb_2*x*/3_□_*x*/3_S for *x* = 0.3, the S atoms in each unit cell **t** are surrounded by six metal (*M*) ions at **t** ± 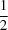 **a, t** ± 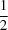 **b** and at **t** ± 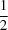 **c**, respectively. Now recall the expression for a composition modulation wave given in equation (1) above, and also that Pauling’s axiom is essentially a six-body correlation function requiring that the average metal stoichiometry around each S atom should be as close as possible to the average composition. In the language of modulated structures, this is equivalent to requiring that
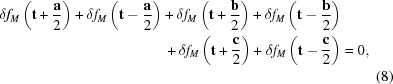
for all average structure unit cells **t**. Substituting equation (1) into equation (8) then gives
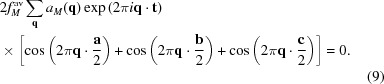
Note that the unit-cell dependent term has been taken to the front of the equation, leaving the term in square brackets. If this term is zero then Pauling’s axiom is satisfied for all unit cells. Finally, substituting **q** = *h* **a** 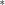 + *k* **b** 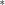 + *l* **c** 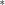 into the above expression, where *h, k* and *l* are here understood to be continuous variables and not integers, gives 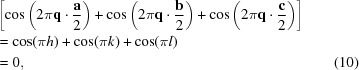
as the relation describing the experimentally observed diffuse distribution. This long-range six-body correlation, which is obeyed for all primitive parent unit cells, corresponds to the long-range order (LRO) encoded in the ordered ‘diffuse’ distribution and is the reason that the diffuse distribution is moderately sharp perpendicular to the continuous diffuse intensity surface.

In fact, many defect NaCl-type solid-solution phases have structured diffuse distributions of essentially the same form, *e.g.* LiFeO_2_ [see Brunel *et al.* (1972 ▶)], transition metal carbide and nitride (*M*C_1−*x*_ and *M*N_1−*x*_) solid-solution phases (Billingham *et al.*, 1972 ▶; Brunel *et al.*, 1972 ▶; Sauvage & Parthé, 1972 ▶; De Ridder *et al.*, 1977 ▶), the wide-range nonstoichiometric (1−*x*)*M* ^2+^S·*x*Ln^3+^_2/3_S (*M* = Mg, Ca or Mn, Ln = a rare earth ion or Y) solid-solution phases (Flahaut, 1979 ▶; Withers *et al.*, 2007 ▶) and even the oxyfluoride K_3_MoO_3_F_3_ [see Withers *et al.* (2003 ▶)]. This suggests that it could perhaps be useful to consolidate the shapes and symmetries of such common highly structured diffuse intensity distributions into some form of atlas, along with the associated real-space implications discussed in this paper. Indeed, Sauvage & Parthé (1974 ▶) have already gone some distance along this route. Likewise, for diffuse intensity in the shape of rods or planes, lists of relevant irreps that might explain systematic extinction conditions could be listed, along with any associated systematic absences (see §3.1 above).

### 3.3. The inherently flexible SiO_2_-tridymite framework structure   

The final example is the widespread family of inherently flexible framework structures (*e.g.* zeotypic microporous molecular sieve materials, nanoporous molecular framework structures, the silica polymorphs and their aluminophosphate analogues *etc.*), as represented here by the case of SiO_2_-tridymite. All such flexible framework structures are built out of corner-connected essentially rigid linkers or polyhedral units, SiO_4_ tetrahedral units in the case of SiO_2_-tridymite. While the constituent polyhedral size and overall topology of such systems may be fixed/constrained, the relative orientations of the polyhedra are not. While it is not *a priori* possible to predict the particular structured diffuse distribution shown in Fig. 2 ▶, nonetheless the lessons learned from the previous two examples remain. The sharpness of the curved diffuse distribution will arise from a LRO constraint that must be obeyed for all parent unit cells **t**, while the SRO (the continuous diffuse surface) will be manifested by differing local polyhedral orientations. The long-range constraint in all such materials is clearly that the constituent polyhedra must all rotate cooperatively as a result of the fact that *e.g.* each oxygen in SiO_2_-tridymite belongs to two distinct SiO_4_ neighbouring tetrahedra and hence can not rotate or displace independently. For beautiful examples of how cooperative rotations are possible in silicates, see the *Crystalline Silica* section of https://www.esc.cam.ac.uk/teaching/mineral-sciences/mineral-physics-movies.

**Figure 7:**
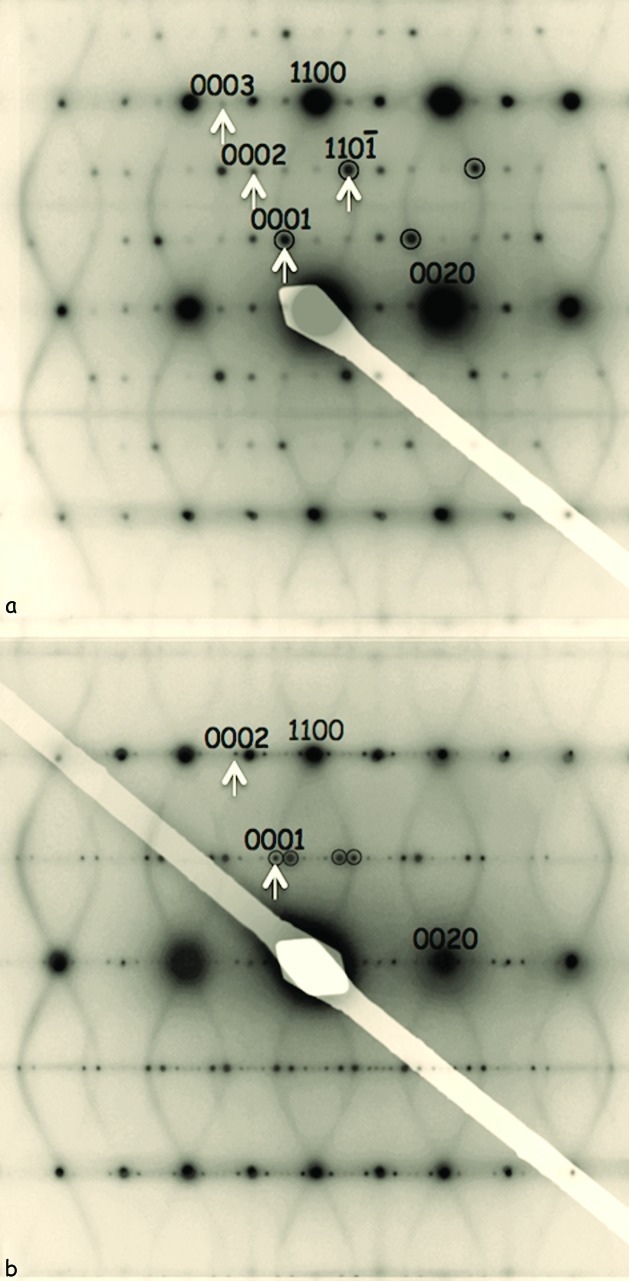
〈
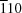
〉 zone-axis EDPs of (*a*) L3-*T*_0_ (MX-1) and (*b*) an incommensurate polymorph of SiO_2_-tridymite. The equivalent 〈
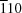
〉 zone-axis EDP of high-temperature SiO_2_-tridymite above 250°C, with its characteristic curved diffuse intensity distribution, is superimposed in order to show that the strongest incommensurate satellite reflections in both cases fall on the characteristic diffuse distribution. Indexing in both cases is with respect to the HP *P*6_3_/*mmc* ideal tridymite parent structure. See Withers *et al.* (1994 ▶) and Withers (2003 ▶) for details.

SiO_2_-tridymite has been chosen as an illustrative example for two reasons. Firstly, its high-temperature reciprocal space features a complex curved diffuse distribution, the shape of which is by no means obvious (see *e.g.* Figs. 1 ▶ *b* and 2 ▶), as well as diffuse lines, planes and points in reciprocal space [see Hammonds *et al.* (1996 ▶) and Dove *et al.* (2000 ▶)] and, secondly, it possesses extraordinary structural polymorphism (see *e.g.* Fig. 7 ▶). At least three distinct room-temperature polymorphic phases, as well as at least five other temperature- and/or stress-dependent polymorphic variant phases, have been reported in the literature [see, for example, Graetsch & Flörke (1991 ▶), Withers *et al.* (1994 ▶), Graetsch (1998 ▶), Hammonds *et al.* (1996 ▶) and Dove *et al.* (2000 ▶), and references contained therein]. Such complex polymorphism indicates a very delicate energetic and/or kinetic balance between the multitude of potential ways of combining the various possible types of correlated rotations and coupled displacements of the ideal (parent) highest temperature H1-*T*_o_ (HP) tri­dymite SiO_4_ tetrahedral framework structure to give lower temperature polymorphic structures.

For example, Fig. 7 ▶ shows 〈
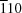
〉 zone-axis EDPs of two of the polymorphs of SiO_2_-tridymite (observed at room temperature), with an equivalent 〈
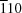
〉 zone-axis EDP of the high-temperature (above 250°C) SiO_2_-tridymite structure superimposed for comparison purposes. (Note that the curved underlying diffuse distribution is not present when the satellite reflections are present. It is put there only for comparison purposes, see below). Indexing of the parent Bragg reflections, **G** = *h* a* + *k* b* + *l* c* + *m* **q** with *m* = 0, in Fig. 7 ▶ is with respect to the highest-temperature H1-*T*_o_ (HP) form of tridymite, space group *P*6_3_/*mmc*. The observed incommensurate satellite reflections occur at **G** + *m* **q** = *h* a* + *k* b* + *l* c* + *m* **q**, where *h, k, l* and *m* are integers and **q** is the relevant incommensurate primary modulation wavevector; **q** = 0.331[a* + b*] − 0.498c* in the case of Fig. 7 ▶(*a*), and 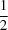
[a* + b*] − 0.608c* in the case of Fig. 7 ▶(*b*). Note that the strongest incommensurate satellite reflections in both cases (labelled with either *m* = 1 or −1 and circled in Fig. 7 ▶) fall precisely on the curved diffuse intensity distribution described by the equation
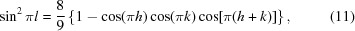
characteristic of the *P*6_3_/*mmc* ideal tridymite parent structure [see Withers (2003 ▶) for details of the relevant constraints used to derive this relationship, and Fig. 2 ▶ for a three-dimensional plot of this equation].

While there is no space here to explain in detail how this equation was derived, the basic principle is simply to write down the allowed tetrahedral rotational and displacive modulations of a particular tetrahedron in a particular parent unit cell, **t**, in a modulation wave form [*i.e.* involving cos(2π**q**·**t**) and sin(2π**q**·**t**) terms], and then take advantage of the fact that any particular O atom belongs to two distinct tetrahedra. Insisting that the local displacement of the independent oxygen ions must be the same for both of these tetrahedra, not only locally but for all unit cells **t**, then leads to the shape of the diffuse distribution given above. Indeed, it also gives the corresponding displacement eigenvector, apart from an overall amplitude and starting phase [see, for example, Fig. 5 of Withers *et al.* (2003 ▶)]. Again, there is a long-range component to the observed diffuse distribution, in that all tetrahedra must rotate and displace cooperatively for all parent unit cells **t**. This is why the diffuse distribution is so sharp perpendicular to its surface. On the other hand, the rotation and displacement of any particular tetrahedron differ from parent unit cell to parent unit cell, giving the continuous short-range ordered part of the diffuse distribution.

**Figure 8:**
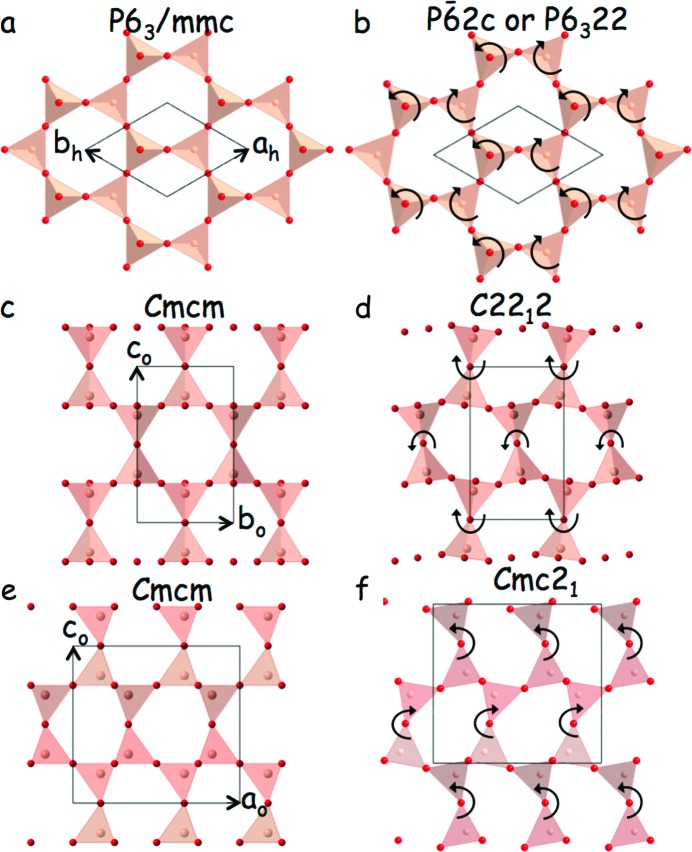
The ideal tridymite parent structure with (*a*) the *P*6_3_/*mmc* setting and in a closely related (*c*), (*e*) orthorhombic *Cmcm* setting, along with three **q** = 0 RUMs of this ideal parent tridymite structure type involving coupled rotations around the parent hexagonal (*b*) **c**_h_ = **c**_0_, (*d*) **a**_h_ − **b**_h_ = **a**_0_ and (*f*) **a**_h_ + **b**_h_ = **b**_0_ directions. Only two tetrahedral layer-thick projections are shown in each case, for clarity. Note that rotation around **a**_0_ and **b**_0_ automatically lowers the parent hexagonal symmetry to orthorhombic (hence the different setting used). In the case of (*b*), again only two (001)_h_ layers are shown. In this case, however, there need be no correlation from one such two-layer slab to the next, which thus gives rise to a line of RUMs along **c**. For **q** = 0, if the other two (001)_h_ layers rotate in phase the resultant space group symmetry is 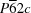. Alternatively, if they rotate out of phase a *P*6_3_22 space group results.

The fact that the primary modulation wavevectors of the two incommensurate polymorphs of SiO_2_-tridymite in Fig. 7 ▶ fall precisely on the curved diffuse distribution described by equation (11) shows that the diffuse distribution(s) characteristic of the higher temperature tridymite phases map out the very low (essentially zero) frequency rigid unit modes (RUMs) of distortion (Dove *et al.*, 2000 ▶) of the ideal tridymite framework structure and hence some of its possible lower temperature modes of distortion. In this context, it is worth pointing out that the characteristic curved type of ‘diffuse’ intensity distribution in Fig. 2 ▶ is only one of several quite distinct types of diffuse distribution that are characteristic of, and indeed can co-exist within, the highest temperature polymorphic phases of SiO_2_-tridymite. Fig. 8 ▶, for example, shows three **q** = 0 RUM modes of the ideal parent tridymite structure type involving coupled rotations around the parent hexagonal directions, Fig. 8 ▶(*b*) **c**_h_ = **c**_0_, Fig. 8 ▶(*d*) **a**_h_ − **b**_h_ = **a**_0_ and Fig. 8 ▶(*f*) **a**_h_ + **b**_h_ = **b**_0_. The ideal unrotated tridymite parent structure is shown in Figs. 8 ▶(*a*), 8 ▶(*c*) and 8 ▶(*e*), respectively. Note that rotation around **a**_0_ and **b**_0_ automatically lowers the parent hexagonal symmetry to orthorhombic. In the case of Fig. 8 ▶(*b*), only two (001)_h_ layers are shown. The other two (001)_h_ layers can either rotate in phase (giving rise to a 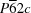 space group) or out of phase (giving rise to a *P*6_3_22 space group).

From the modulation wave point of view, note that, while the strongest primary incommensurate satellite reflections in both of the above cases fall precisely on the curved diffuse intensity distribution in Fig. 7 ▶, the second- and third-order satellite reflections in both cases (labelled with either 2, 3 or 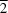 in the fourth index position) do *not* fall on the curved diffuse intensity distribution. By contrast, only the first-order harmonic, or primary, **G** ± **q** satellite reflections, where **q** is defined to be the continuous points on the curved diffuse surface, are present in Fig. 1 ▶(*b*), *i.e.* again no second-harmonic (*m* = 2) copy of the primary diffuse distribution is visible in Fig. 1 ▶(*b*).

For further examples of this phemonenon, see *e.g.* Fig. 4 of Withers (2008 ▶). Such observations strongly suggest that there is almost invariably never any correlation between the different individual primary modulation wavevectors constituting the primary diffuse distribution, in contrast with what is clearly the case for a conventional (3+*n*)-dimensional (*n* > 1) incommensurately modulated structure (see *e.g.* Figs. 3 ▶ *b* and 6 ▶), where higher-order harmonic satellite reflections are clearly present. Thus, in almost all cases an eminently reasonable assumption is that the generally weak primary diffuse distribution at **G** ± **q** in modulated structures of this ordered ‘disorder’ type arises solely from compositional and/or displacive modulation waves associated with the different individual primary modulation wavevectors **q**.

## 4. Some conclusions   

Bernal’s aphorism that ‘…crystallization is death…’ (Mackay, 1969 ▶) needs expanding. Cystallization with *only* pure point diffraction/sharp Bragg reflections is death! Luckily, there are plenty of somewhat free materials, of which the above examples barely scratch the surface, exhibiting not only sharp Bragg reflections but also highly structured characteristic diffuse intensity distributions that simultaneously encode both truly SRO and significantly longer range order. We should work to understand the relevant (hierarchical) rules that give rise to structured diffuse distributions and their shapes. Likewise, if diffuse shapes are simple, *e.g.* one-dimensional rods or two-dimensional planes, diffuse distributions can and do exhibit characteristic extinction conditions. We should systematically look for them, document them and use them, wherever appropriate! Finally, why use only use pair distribution functions when you can use multi-body correlations. Nature appears to!

## References

[bb1] Atkins, P. M. W., Overton, T., Rourke, J., Weller, M. & Armstrong, F. (2010). *Shriver and Atkins’ Inorganic Chemistry*, 5th Ed., p. 66. Oxford University Press.

[bb2] Baake, M. & Grimm, U. (2009). *Phys. Rev. B*, **79**, 020203.

[bb3] Baake, M. & Grimm, U. (2012). *Chem. Soc. Rev.* **41**, 6821–6848. 10.1039/c2cs35120j 22797147

[bb4] Billingham, J., Bell, P. S. & Lewis, M. H. (1972). *Acta Cryst.* A**28**, 602–606.

[bb5] Bradley, C. J. & Cracknell, A. P. (1972). *The Mathematical Theory of Symmetry in Solids.* Oxford University Press.

[bb6] Brink, F., Withers, R. L. & Thompson, J. G. (2000). *J. Solid State Chem.* **155**, 359–365.

[bb7] Brouns, E., Visser, J. W. & de Wolff, P. M. (1964). *Acta Cryst.* **17**, 614.

[bb8] Brown, I. D. (2006). *The Chemical Bond in Inorganic Chemistry: The Bond Valence Model.* Oxford University Press.

[bb9] Brunel, M., De Bergevin, F. & Gondrand, M. (1972). *J. Phys. Chem. Solids*, **33**, 1927–1941.

[bb10] De Ridder, R., Van Dyck, D., Van Tendeloo, G. & Amelinckx, S. (1977). *Phys. Status Solidi. A*, **40**, 669–683.

[bb11] Dove, M. T., Pryde, A. K. A. & Keen, D. A. (2000). *Miner. Mag.* **64**, 267–283.

[bb12] Fisher, I. R., Islam, Z., Panchula, A. F., Cheon, K. O., Kramer, M. J., Canfield, P. C. & Goldman, A. I. (1998). *Philos. Mag. B*, **77**, 1601–1615.

[bb13] Flahaut, J. (1979). *Handbook on the Physics and Chemistry of the Rare Earths*, edited by K. L. Gschneider and L. Eyring, Vol. 4, ch. 31, pp. 1–88. Amsterdam: North Holland.

[bb14] Graetsch, H. (1998). *Am. Miner.* **83**, 872–880.

[bb15] Graetsch, H. & Flörke, O. W. (1991). *Z. Kristallogr.* **195**, 31–48.

[bb16] Hammonds, K. D., Dove, M. T., Giddy, A. P., Heine, V. & Winkler, B. (1996). *Am. Mineral.* **81**, 1057–1079.

[bb17] Herpin, A., Mériel, P. & Villain, J. (1960). *J. Phys. Radium*, **21**, 67–72.

[bb18] Ishimasa, T., Nissen, H.-U. & Fukano, Y. (1985). *Phys. Rev. Lett.* **55**, 511–513. 10.1103/PhysRevLett.55.511 10032372

[bb19] Janner, A. (1997). *Acta Cryst.* A**53**, 615–631.

[bb20] Janner, A. (2001). *Cryst. Eng.* **4**, 119–129.

[bb21] Janner, A. & Janssen, T. (1977). *Phys. Rev. B*, **15**, 649–658.

[bb22] Janssen, T. (1986). *Acta Cryst.* A**42**, 261–271.

[bb23] Janssen, T. (2013). *Aperiodic Crystals*, pp. 1–9. Dordrecht: Springer Press.

[bb24] Janssen, T., Janner, A., Looijenga-Vos, A. & de Wolff, P. M. (1995). *International Tables for Crystallography*, Vol. C, edited by A. J. C. Wilson, pp. 797–835. Heidelberg: Springer.

[bb25] Johnson, C. K. & Watson, C. R. (1976). *J. Chem. Phys.* **64**, 2271–2286.

[bb26] Ling, C. D., Schmid, S., Blanchard, P. E. R., Petříček, V., McIntyre, G. J., Sharma, N., Maljuk, A., Yaremchenko, A. A., Kharton, V. V., Gutmann, M. & Withers, R. L. (2013). *J. Am. Chem. Soc.* **135**, 6477–6484. 10.1021/ja3109328 23570580

[bb27] Ling, C. D., Withers, R. L., Schmid, S. & Thompson, J. G. (1998). *J. Solid State Chem.* **137**, 42–61.

[bb28] Mackay, A. L. (1969). *Chimia*, **23**, 433–437.

[bb29] Mackay, A. L. & Finney, J. L. (1973). *J. Appl. Cryst.* **6**, 284–289.

[bb30] Perez-Mato, J. M., Madariaga, G. & Tello, M. J. (1986). *J. Phys. C Solid State Phys.* **19**, 2613–2622.

[bb31] Perez-Mato, J. M., Madariaga, G., Zuñiga, F. J. & Garcia Arribas, A. (1987). *Acta Cryst.* A**43**, 216–226.

[bb32] Pouget, J. P., Shirane, G., Hastings, J. M., Heeger, A. J., Miro, N. D. & MacDiarmid, A. G. (1978). *Phys. Rev. B*, **18**, 3645–3656.

[bb33] Sauvage, M. & Parthé, E. (1972). *Acta Cryst.* A**28**, 607–616.

[bb34] Sauvage, M. & Parthé, E. (1974). *Acta Cryst.* A**30**, 239–246.

[bb46] Shechtman, D., Blech, I., Gratias, D. & Cahn, J. W. (1984). *Phys. Rev. Lett.* **53**, 1951–1954.

[bb35] Steurer, W. (2012). *Chem. Soc. Rev.* **41**, 6719–6729.

[bb36] Urones-Garrote, E., Gómez-Herrero, A., Landa-Cánovas, Á. R., Withers, R. L. & Otero-Díaz, L. C. (2005). *Chem. Mater.* **17**, 3524–3531.

[bb37] Welberry, T. R. & Butler, B. D. (1994). *J. Appl. Cryst.* **27**, 205–231.

[bb38] Welberry, T. R., Withers, R. L. & Mayo, S. C. (1995). *J. Solid State Chem.* **115**, 43–54.

[bb39] Withers, R. L. (2003). *Solid State Sci.* **5**, 115–123.

[bb40] Withers, R. L. (2008). *Advances in Imaging and Electron Physics*, edited by P. W. Hawkes, Vol. 152, ch. 6, pp. 303–337. Amsterdam: Elsevier.

[bb41] Withers, R. L., Aroyo, M. I., Perez-Mato, J. M. & Orobengoa, D. (2010). *Acta Cryst.* B**66**, 315–322. 10.1107/S0108768110009031 20484802

[bb42] Withers, R. L., Thompson, J. G., Xiao, Y. & Kirkpatrick, R. J. (1994). *Phys. Chem. Miner.* **21**, 421–433.

[bb43] Withers, R. L., Urones-Garrote, E. & Otero-Diaz, L. C. (2007). *Philos. Mag.* **87**, 2807–2813.

[bb44] Withers, R. L., Welberry, T. R., Brink, F. J. & Norén, L. (2003). *J. Solid State Chem.* **170**, 211–220.

[bb45] Wolff, P. M. de (1977). *Acta Cryst.* A**33**, 493–497.

